# Inpatient Management of Encephalopathy

**DOI:** 10.7759/cureus.22102

**Published:** 2022-02-10

**Authors:** Pramod Reddy, Kaleb Culpepper

**Affiliations:** 1 Internal Medicine, University of Florida College of Medicine, Jacksonville, USA; 2 Neurology, University of Florida College of Medicine, Jacksonville, USA

**Keywords:** bickerstaff brainstem encephalitis, acute disseminated encephalomyelitis, primary cns vasculitis, nmda-r encephalitis, hashimoto encephalopathy, paraneoplastic encephalitis, autoimmune encephalitis

## Abstract

Unexplained encephalopathy is a common occurrence in tertiary care centers and neurologic disorders should be considered after ruling out the infectious, toxic and metabolic etiologies. Neuroimaging combined with a thorough history and examination is often helpful in ruling out stroke and fulminant demyelinating encephalopathies. Autoimmune encephalopathy should be suspected in any patient with unexplained acute or subacute onset encephalopathy or rapidly progressing dementia. Anti-N-methyl-D-aspartate receptor (NMDA-R) encephalitis is the most studied form and Hashimoto encephalitis is the most controversial form of autoimmune encephalopathies. Obtaining a combined serum and Cerebrospinal fluid (CSF) autoantibody testing will increase the diagnostic yield of autoimmune and paraneoplastic encephalitis. When diagnosing NMDA receptor antibodies CSF is always more sensitive than serum and in contrast, voltage-gated potassium channel (VGKC) complex antibodies are more readily detectable in serum than in CSF. Neural-specific antibody tests frequently result after several weeks and treatment should be administered without a significant delay to prevent brain damage. Autoimmune encephalitis is often treatment responsive when immunotherapy (glucocorticoids, intravenous immune globulin, plasma exchange) is used in various combinations. The absence of inflammatory markers and autoantibodies in the serum or CSF may not rule out the possibility of paraneoplastic encephalopathies.

## Introduction and background

Altered mental status (AMS) is a common occurrence in both hospitalized and patients visiting the emergency department. Severe encephalopathy results in AMS due to global brain dysfunction and manifests with headache, nausea, vomiting, visual disturbances, confusion, seizures followed by stupor and coma in advanced cases. Acute encephalopathy can occur from both systemic and neurological processes and patients require rapid evaluation and intervention in order to limit the brain injury. Examples of systemic causes include drug overdose, drug withdrawal, electrolyte disturbances, thyroid disorders, hypoxia, hypoglycemia, hypotension, severe hypertension and organ (renal/hepatic) failure. Focal central nervous system (CNS) derangements include tumors, edema with mass effect, seizures, stroke, bleeding, infectious meningoencephalitis and various forms of demyelinating, vasculitis and autoimmune encephalopathies. While minor focal deficits may be present on the neurologic examination in patients with metabolic encephalopathies, the finding of prominent focal signs should suggest the possibility of a structural lesion.

Background

It is important for physicians to be aware of the various subtypes of encephalitis presentations in patients with both autoimmune and paraneoplastic encephalitis. Limbic encephalitis refers to an inflammatory process localized to structures of the limbic system (e.g., hippocampus, amygdala, hypothalamus, cingulate gyrus, limbic cortex) and is characterized by acute or subacute mood and behavioral changes, short-term memory problems, focal seizures with impaired awareness and cognitive dysfunction [1]. Brainstem encephalitis is characterized by extraocular movement impairment, opsoclonus, nystagmus, dysphagia, dysarthria, sensorineural deafness and vertigo. The term rhombencephalitis refers to inflammation affecting the lower brainstem and cerebellum [2]. Encephalomyelitis characteristically involves the temporal-limbic regions, brainstem, cerebellum, spinal cord, dorsal root ganglia and autonomic nervous system [3].

## Review

It is important to clinically distinguish acute encephalopathy from delirium, posterior reversible encephalopathy syndrome (PRES), nonconvulsive status epilepticus (NCSE) and Creutzfeldt-Jakob disease (CJD) which can be challenging in some cases.

Delirium

Delirium (acute confusional state) develops over hours to days and often presents with subtle changes in the level of awareness with nocturnal worsening of symptoms. Patients often appear distracted during conversations and an altered level of consciousness with fluctuations is evident in more advanced cases. In nearly one-half of older patients presenting with delirium, the preexisting conditions that increase the risk include dementia, stroke and Parkinsonism. The other common factors that may precipitate delirium include polypharmacy, infection, dehydration, immobility, malnutrition and the use of bladder catheters. In some cases, the first presentation of delirium in an elderly patient may represent unrecognized underlying dementia [4]. In addition to treating the underlying acute illness responsible for delirium, other effective measures include early mobilization and minimizing the use of physical restraints. Short-term use of psychotropic medications (e.g., haloperidol, quetiapine and risperidone) should be reserved for treatment of severe agitation or psychosis. Benzodiazepines should be avoided in patients with delirium, except in cases of sedative drug and alcohol withdrawal. Empiric parenteral thiamine supplementation should be considered in all patients with delirium (e.g., 500mg IV every 8hrs for three days) to treat possible Wernicke encephalopathy [5]. Thiamine supplementation is safe, inexpensive and rapid clinical improvement can be seen in patients with alcoholism, gastric-bypass surgery and malnourishment.

Posterior reversible encephalopathy syndrome

PRES most often occurs in the setting of hypertensive crisis, sepsis, renal failure, autoimmune diseases, preeclampsia or with cytotoxic immunosuppressive therapy (cyclosporine, gemcitabine, tacrolimus, bevacizumab). The typical clinical syndrome includes headache, confusion, visual disturbances, altered consciousness and tonic-clonic seizures. Physical examination findings in some patients may reveal limb weakness, brisk deep tendon reflexes and Babinski signs [6]. Neuroimaging is essential to the diagnosis of PRES and in most patients, CT/MRI findings are consistent with vasogenic edema predominantly localized to the posterior cerebral hemispheres. The distribution of radiologic abnormalities is usually not confined to a single vascular territory and once the acute stroke is excluded, 10%-25% reduction of blood pressure should be attempted by using easily titratable parenteral agents. Discontinuation of offending chemotherapy agents and treatment of other acute medical conditions (e.g., sepsis, renal failure). Antiseizure medications are administered to patients with seizures, which can be discontinued after the clinical resolution.

Nonconvulsive status epilepticus

NCSE is defined as status epilepticus without prominent motor symptoms without full recovery of consciousness between the attacks. While the majority of patients have a prior history of seizures or epilepsy, new-onset NCSE can occur in critically ill hospitalized patients with CNS or systemic illness. Drug-induced NCSE is an important consideration in patients being treated with antibiotics (cefepime, quinolones) and chemotherapy (ifosfamide, cisplatin, busulfan). NCSE diagnosis requires a high index of suspicion and continuous EEG (up to 72 hours) monitoring should be ordered to rule out the condition. Continuous EEG is threefold more sensitive when compared to spot EEGs [7]. NCSE not only responds to traditional intravenous antiepileptic medications (benzodiazepines, levetiracetam or phenytoin) but also to corticosteroids, suggesting an inflammatory process in the pathogenesis. Refractory NCSE typically requires continuous infusions of midazolam, propofol, ketamine or pentobarbital in the intensive care unit and is associated with a high mortality rate (up to 50%) due to a combination of primary underlying acute process and secondary neuronal injury from prolonged seizure activity [8].

Creutzfeldt-Jakob disease

CJD is the most common prion disease and presents with a rapidly progressive mental deterioration often associated with behavioral abnormalities, extrapyramidal signs and startle myoclonus. MRI brain typically shows the abnormal hyperintense signals in the putamen, head of the caudate and cortex. EEG finding of periodic sharp wave complexes (PSWC) has a low sensitivity but high specificity for the diagnosis of CJD. Cerebrospinal fluid (CSF) 14-3-3 protein test is nonspecific in patients with CJD, but RT-QuIC (real-time quaking-induced conversion) in CSF is a very sensitive and specific diagnostic test [9].

Immunotherapy responsive encephalopathies

**Figure 1:**
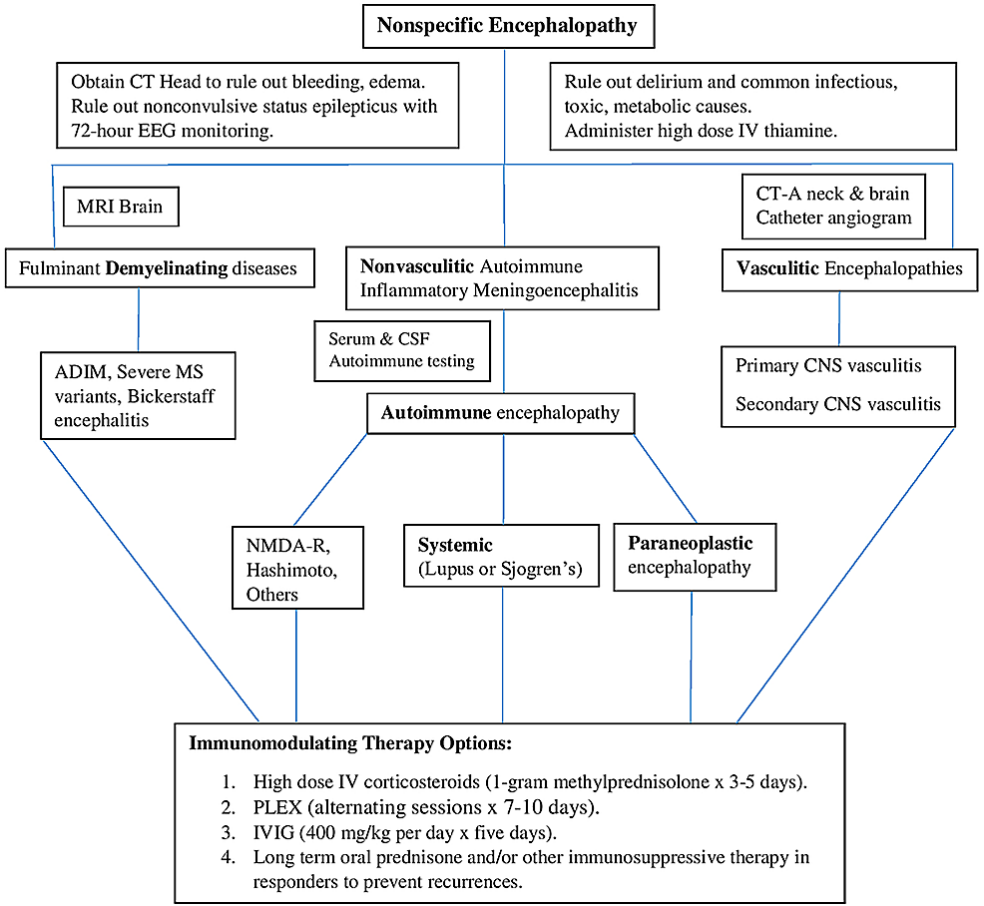
Workup algorithm of nonspecific encephalopathy

The etiology of immunotherapy responsive encephalopathies is diverse (Table 1) and it is useful to divide them into demyelinating, vasculitic and non-vasculitic causes for easier understanding and management (Figure 1).

Fulminant Demyelinating Diseases

Severe relapses of MS and its Marburg variant can present with acute encephalopathy along with other demyelinating etiologies like acute disseminated encephalomyelitis (ADEM), Bickerstaff brainstem encephalitis (BBE) and neuromyelitis optica (NMO) spectrum disorders.

ADEM is an autoimmune disorder that is triggered by an environmental stimulus (e.g., viral infections in 75% cases) in genetically susceptible children (average age of onset between three to seven years). Patients present with rapidly developing encephalopathy along with neurological signs of hemiparesis, cerebellar ataxia, optic neuritis and myelopathy. The demyelination mainly affects the white matter of the brain, brainstem and spinal cord. Hyperacute hemorrhagic variants of ADEM also reveal multifocal white matter hemorrhages. MRI findings may not appear in some cases for several weeks after the onset of ADEM [10]. Serum testing for the myelin oligodendrocyte glycoprotein (MOG) IgG autoantibody and the aquaporin-4 (AQP4) IgG autoantibody should be done in patients presenting with suspected ADEM. The MOG antibody disease is both a potential cause of ADEM and other CNS demyelinating disorders. The anti-AQP4 IgG antibody is a specific biomarker for NMO spectrum disorder.

BBE is characterized by encephalopathy, hyperreflexia, ophthalmoplegia and ataxia, typically preceded by upper respiratory or gastrointestinal infections [11]. BBE is associated with anti-GQ1b antibodies and together with Guillain-Barre syndrome and Miller-Fisher syndrome, it forms a spectrum of post-infectious demyelinating diseases.

Vasculitic encephalopathies

CNS vasculitis results in inflammation of the small and medium blood vessels of the brain, spinal cord, and meninges. CNS vasculitis is considered secondary when it occurs in patients with systemic autoimmune diseases (e.g., lupus) and vasculitides, which include antineutrophil cytoplasmic antibody (ANCA) associated (granulomatosis with polyangiitis) conditions to ANCA negative disorders like polyarteritis nodosa. Encephalopathy in secondary CNS vasculitis results from the involvement of cerebral vessels and stroke. Rarely secondary CNS vasculitis also occurs from infectious processes like varicella-zoster vasculopathy.

Primary CNS vasculitis is defined by inflammation of the cerebral vasculature without affecting the other organs [12]. It is characterized by a long prodromal period, but few patients can present with acute encephalitis. The CSF is abnormal in the majority of patients with nonspecific elevation of protein and lymphocytic pleocytosis. MRI of the brain commonly shows multiple infarcts in several vascular territories and conventional catheter-based angiography can detect segmental narrowing in multiple vessels. A leptomeningeal biopsy can confirm the diagnosis in 75% of patients and patients typically respond to a combination of high-dose glucocorticoids and cyclophosphamide [13].

Nonvasculitic Autoimmune Inflammatory Meningoencephalitis (NAIM)

Autoimmune encephalopathy symptoms typically evolve over days to weeks, but it is not uncommon to see acute presentations. These patients may also have a background of systemic autoimmune diseases or first-degree relatives affected by them. Autoimmune encephalitis syndromes may occur in the presence or absence of cancer and manifest clinically as limbic or brainstem encephalitis. Limbic encephalitis commonly presents with personality changes as well as delirium and focal seizures of temporal origin. Limbic encephalitis is also described in systemic autoimmune conditions without associated secondary CNS vasculitis like hypereosinophilic syndrome, lupus and Sjogren’s syndrome [14].

Several different autoimmune encephalopathies (e.g., anti-LGI1 and mGluR1 encephalitides) have been described in the literature (Table 1) and some of them will be briefly reviewed below.

**Table 1:** Immunotherapy responsive encephalopathies SCLC, small cell lung carcinoma; ANCA, antineutrophil cytoplasmic antibodies; MOG, Anti-myelin oligodendrocyte; VGKC, voltage-gated potassium channel complex; LGI1, leucine-rich glioma inactivated 1; CASPR2/3, contactin-associated protein-2; NMDA, N-methyl-D-aspartate; GABA A/B, gamma-aminobutyric acid; GAD65, glutamic acid decarboxylase, 65 isoform; AMPA, α-amino-3-hydroxy-5-methyl-4-isoxazolepropionic acid; ANNA, antineuronal nuclear antibody; AGNA, anti-glial nuclear antibody; PCA, Purkinje cell cytoplasmic antibody; CRMP-5, collapsin-response mediator-protein 5; NMO-IgG, neuromyelitis optica IgG; GluR3, Glutamate receptor 3; DPPX, dipeptidyl-peptidase–like protein 6.

Condition	Associated findings	Diagnostic studies
Secondary CNS vasculitis	SLE, Sjögren's syndrome, antiphospholipid antibody syndrome, scleroderma, Behçet's disease, polyarteritis nodosa, Wegener's granulomatosis, microscopic polyangiitis, Churg-Strauss syndrome and cryoglobulinemia.	ANA, dsDNA, Anti-Scl-70, ANCA, RF, CCP, APL, SS-A/B, biopsy.
Primary CNS vasculitis	Headache, strokes seen in MRI; MRA/CTA cerebral vessel occlusion.	Meningeal biopsy
Fulminant Demyelinating Diseases	Acute demyelinating encephalomyelitis (ADIM), Bickerstaff brainstem encephalitis (BBE), severe relapses of multiple sclerosis (MS), Marburg MS variant, neuromyelitis optica (NMO)-spectrum disorders.	Anti-MOG in ADIM. Aquaporin in NMO. Anti-GQ1b in BBE.
Neurosarcoidosis	Erythema nodosum, hilar adenopathy or uveitis.	MRI findings; Biopsy
Interferonopathies	Aicardi-Goutieres syndrome - skin vasculitis affecting areas exposed to cold (finger, nose, external ears), interstitial lung disease	MRI - calcification of the basal ganglia.
Hemophagocytic lymphohistiocytosis	Multiorgan involvement with fever, hepatosplenomegaly, lymphadenopathy, neurologic symptoms, very high ferritin and cytopenias.	Infiltration of the bone marrow by macrophages.
IgG4-related disease	Other organs affected (pancreas, bile ducts, salivary/lacrimal glands.	Elevated serum IgG4.
Susac syndrome	Autoimmune-mediated occlusions of microvessels in the brain, retina and inner ear; Branch retinal artery occlusion.	MRI: lesions in corpus callosum
Rasmussen encephalitis	Unilateral inflammation of the cerebral cortex, drug-resistant epilepsy, and progressive neurological and cognitive deterioration.	GluR3
Morvan syndrome	Neuromyotonia with dysautonomia, insomnia and neuropathic pain.	VGKC & CASPR2
NMDA receptor encephalopathy	Children and young adults affected with seizures and behavioral changes; Ovarian teratoma in >50% adult females.	NMDA receptor antibody
Hashimoto encephalopathy	Acute to subacute onset of confusion with alteration of consciousness; Seizures and myoclonus occur commonly;	Thyroid peroxidase, thyroglobulin, enolase.
Various other causes of autoimmune encephalopathies	Anti-IgLON5 disease	IgLON5
	Anti-mGluR1 encephalitis (cerebellar ataxia and cognitive changes)	Anti-mGluR1
	Anti-neurexin-3 alpha encephalitis (orofacial dyskinesias)	Neurexin-3 alpha
	Anti-LGI-1 encephalitis, Thymoma	LGI-1
	Glial fibrillary acidic protein (GFAP) encephalitis	GFAP
Paraneoplastic encephalopathies	SCLC, thymoma	ANNA-1 (Hu)
	SCC, thymoma, Adenocarcinoma (prostate & breast)	VGKC complex
	SCLC, Breast adenocarcinoma	ANNA-2 (Ri)
	SCLC, adenocarcinoma (lung or esophagus)	ANNA-3
	Ovarian and breast carcinoma	PCA-1
	SCLC	PCA-2
	SCLC, Breast adenocarcinoma	Amphiphysin IgG
	SCLC, thymoma	CRMP-5 IgG
	SCLC, NHL, breast & G.I cancer	Ma1 & Ma2
	Testicular germ cell tumors	Ma2
	Thymoma, renal cell, adenocarcinoma (breast/ colon)	GAD65 IgG
	SCLC	AGNA (SOX1)
	SCLC, neuroendocrine neoplasia	GABA A/B receptor
	Cancers of Breast, lung and thymus	AMPA receptor
	Hodgkin lymphoma (Ophelia syndrome) or SCLC	Anti-mGluR5
	B-cell neoplasms, thymoma	Anti-GlyR
	Adenocarcinoma, renal cell carcinoma, lymphoma	Acetylcholine receptor
	B-cell neoplasms	DPPX antibody

Anti-N-methyl-D-aspartate receptor (NMDA-R) encephalitis

NMDA-R encephalitis primarily affects children, young adults and presents with rapid onset of four of the following six features: cognitive dysfunction or abnormal psychiatric behavior, speech dysfunction, seizures, movement disorder, decreased level of consciousness and dysautonomia. Workup usually reveals abnormal EEG, pleocytosis or oligoclonal bands in CSF and elevated inflammatory markers in serum. MRI is often normal or shows transient fluid-attenuated inversion recovery (FLAIR) or contrast-enhancing abnormalities in cortical (brain, cerebellum) or subcortical (hippocampus, basal ganglia, white matter) regions. When performed, positron emission tomography (PET) shows a characteristic increase in the frontal-occipital gradient of cerebral glucose metabolism, which correlates with disease severity. The diagnosis is confirmed by the detection of IgG antibodies to the GluN1 (NR1) subunit of the NMDA receptor in the CSF (serum is less reliable), which is sensitive and specific for the condition [15]. Post-herpes simplex virus encephalitis relapses can be associated with NMDA-R antibodies [16]. Female patients older than 18 years have an associated ovarian teratoma in around 50% of cases and surgical resection is warranted when present. The titer of CSF antibodies correlates more closely with the clinical outcome than serum titers and recovery can be slow (up to two years) despite successful treatment.

Hashimoto encephalopathy (HE)

HE is also referred to as steroid-responsive encephalopathy associated with autoimmune thyroiditis (SREAT). HE occurs more commonly in females (4:1 ratio) and presents with acute onset confusion, psychosis (visual hallucinations/ delusions), altered level of consciousness, seizures (focal/generalized/status epilepticus), myoclonus (40%), stroke-like pattern (25%), diffuse hyperreflexia/other pyramidal tract signs (85%) or as a fulminant pattern with rapid deterioration to coma.

HE is characterized by high levels of either anti-thyroid antibodies (thyroperoxidase, thyroglobulin, thyrotropin receptor) or antibodies against the amino-terminal of α-enolase (present in thyroid and brain tissue). Any single one of these antibodies can be elevated without the others and it is useful to send them all together. These antithyroid antibodies can also be measured in the CSF (without being present in serum in some cases) suggesting thecal production [17]. There is no direct correlation between thyroid antibody titers and the HE disease severity and the incidental finding of anti-thyroid antibodies in the general population (5-20%) should be considered. Higher cut-off levels (more than five times the normal value) have been suggested to differentiate the disease from the false-positive low titer anti-thyroid antibodies in the healthy population. But, patients with a smaller elevation in antibody titers may still respond to steroid challenges in clinical practice [18].

Elevation in serum anti-thyroid antibodies is required for the diagnosis, but the condition is unrelated to Hashimoto thyroiditis. In most cases, there is no clinical or biochemical evidence of thyroid dysfunction, but few patients have underlying hypo or hyperthyroidism. Treated HE patients may also develop clinical Hashimoto thyroiditis years after the diagnosis. Brain MRI is normal in one-half cases and shows nonspecific generalized cerebral atrophy in other cases. The thyroid antibody levels may or may not decrease after the treatment and should not be used to guide therapy [19]. Hashimoto encephalitis remains a diagnosis of exclusion and is underdiagnosed in clinical practice.

Paraneoplastic encephalopathies

Paraneoplastic encephalopathies (comprise around 25% of cases) are caused by an immunologic response directed against shared antigens that are expressed by both the tumor and the nervous system. Paraneoplastic encephalitis manifests in most cases as limbic or brainstem encephalitis, but in some cases as myelitis (involvement of the dorsal root ganglia) or encephalomyelitis (involvement of the temporal-limbic regions, brainstem, cerebellum, spinal cord, dorsal root ganglia and autonomic nervous system).

The most frequent neoplasms associated with paraneoplastic encephalitis are small cell lung cancer (75% cases), testicular tumors, thymoma, ovarian tumors, breast cancer, and Hodgkin lymphoma. Antibodies can be detected in the serum or CSF and individual cancers with correlative antibodies are listed in Table 1. In more than half of patients, paraneoplastic encephalopathy may precede the diagnosis of underlying malignancy [20].

Workup of encephalopathy

Standard laboratory evaluation of acute encephalopathy patients should include assessment of serum electrolytes, thyroid/kidney/liver function tests, complete blood count, coagulation assays and infectious workup (Table 2). C-reactive protein (CRP) and the erythrocyte sedimentation rate (ESR) are elevated in several patients with inflammatory encephalopathies. But, the absence of systemic inflammatory markers may not rule out intracerebral inflammatory conditions.

**Table 2:** Laboratory investigations in nonspecific encephalopathy and rapid onset dementia

Initial diagnostic workup	Subsequent diagnostic workup	Autoimmune & neural-specific antibody testing
Complete blood count (CBC), prothrombin time (PT), partial thromboplastin time (PTT), erythrocyte sedimentation rate (ESR), C-reactive protein, renal function panel, liver enzymes, thyroid function tests, vitamin B12, calcium and ammonia levels.	MRI/MRA to exclude vascular disease, demyelination and tumors. CT angiogram of neck and cerebral vessels to rule out vasculitis. Leptomeningeal biopsy in suspected cases of primary CNS vasculitis.	Antithyroid antibodies: Antibodies to thyroid peroxidase (TPO), and thyroglobulin, thyrotropin receptor (TRAb). Anti-NH2-terminal of α-enolase (NAE) antibodies.
HIV, hepatitis and syphilis screen.	Continuous EEG to rule out nonconvulsive status epilepticus.	Systemic autoimmune workup: Antinuclear antibodies (ANA), double-stranded DNA (anti-dsDNA) antibodies, antineutrophil cytoplasmic antibodies (ANCA), anti ribonucleoprotein (anti-RNP), anti-Sjogren (anti-SSA, anti-SSB), anti-Smith, anti-Scl-70 antibodies, rheumatoid factor(RF) and anti-cyclic citrullinated peptide (CCP).
Urinalysis, chest x-ray and blood cultures. CT head to rule out bleeding and cerebral edema. Serum and urine toxicology screen and lead and heavy metal poisoning screen. Serum viscosity, C3, C4 and IgG4 level as needed.	Lumbar Puncture fluid additional tests: Markers of elevated intrathecal IgG synthesis (myelin based protein, oligoclonal bands, IgG index, and synthesis rate). CSF 14-3-3 protein (for possible Creutzfeldt-Jakob disease).	
Lumbar Puncture: Cell count and culture to rule out bacterial, viral and fungal infections. Save fluid from the lumbar puncture for additional tests as needed.	CT scan of the chest, abdomen and pelvis to rule out mass lesions. Testicular ultrasound, mammogram, pelvic ultrasound, endoscopy, PET/CT scan as needed.	Serum & CSF paraneoplastic and autoantibody panels; Mayo clinic laboratories test panels ID: PAVAL, ENC2 and FGAGM (Ganglioside (Asialo-GM1, GM1, GM2, GD1a, GD1b, and GQ1b) Antibodies).

All patients with AMS of unknown etiology should undergo computed tomography (CT) scanning of the brain to rapidly rule out hemorrhage, hydrocephalus and edema with midline shift. In patients with nondiagnostic initial workup, a lumbar puncture (LP) should be done to exclude infectious or inflammatory processes (Table 2). Older patients with meningitis are more likely to present with AMS rather than the classic triad of fever, headache and meningismus. After ruling out infectious, toxic and metabolic causes of encephalopathy, neurology consultation should be requested for further work up of nonspecific encephalopathy [21].

In patients with nonspecific encephalopathy magnetic resonance imaging (MRI) may provide more specific information in patients suspected of acute ischemic stroke, demyelinating and posterior fossa lesions. MR angiography (MRA) and MR venography (MRV) of the brain are helpful to rule out vascular pathologies (e.g., basilar artery thrombosis). It is important to perform MR studies under sedation to prevent motion artifacts in patients with AMS. EEG in most patients with acute encephalopathy shows diffuse bilateral background slowing with a posterior dominant rhythm in low alpha, theta or delta range with or without delta or theta bursts. Triphasic waves are a common but nonspecific finding in several toxic and metabolic encephalopathies.

CSF analysis is abnormal in most autoimmune encephalopathy patients with elevated protein concentration and pleocytosis. Physicians also commonly order CSF markers of CNS inflammation like myelin basic protein (MBP), oligoclonal bands (OCBs) and IgG index. MBP is a measure of fragments released into the spinal fluid as a result of the myelin breakdown from demyelinating diseases (e.g., multiple sclerosis, MS). OCBs are immunoglobulins generated by plasma cells specific to the CNS compartment and presence of two or more oligoclonal bands suggests the intrathecal production of IgG. CSF IgG index refers to the proportion of IgG in reference to the total protein in CSF and a positive test is considered to be greater than 12% of the total protein. While these markers are useful in the diagnosis of MS, their utility is limited in autoimmune encephalopathies, where nonspecific elevations are seen due to CNS inflammation.

Autoantibody testing of the serum and CSF can be ordered to assess several autoimmune and paraneoplastic encephalopathies based on clinical suspicion. When evaluating encephalitis of unknown origin in younger patients, the assays that are ordered in our institution include CSF NMDA Receptor, GABA-A, GABA-B, VGKC, AMPA and Aquaporin-4 Receptor IgG antibodies. For elderly patients with suspicion of cancer, the workup also includes PCCA/ANNA, neuronal nuclear antibodies (Hu, Ri, Yo) and Purkinje cell IgG antibodies. Further comprehensive autoimmune encephalopathy workup can be ordered in the serum (PAVAL) and CSF (ENC2) by the Mayo Clinic (refer to Tables 1, 2).

Immunomodulating therapy of autoimmune encephalopathy

It is important to promptly treat autoimmune encephalopathies, as early initiation of treatment has been shown to improve outcomes and reduce the risk of relapses. Systemic autoimmune workup (e.g., ANA and ANCA) and anti-thyroid antibodies usually result in a few days, but the results of other specific autoimmune and paraneoplastic antibody panels are not available for several weeks.

Steroid Challenge

A challenge with high-dose corticosteroids is the mainstay of therapy in patients with suspected autoimmune encephalopathy. Intravenous methylprednisolone 1,000mg for three to five days is commonly used and the clinical response can be dramatic in several patients. In steroid responders, oral prednisone can be started with an initial dose of 1mg/kg/day (based on the ideal body weight). Steroid refractory cases are treated with either plasmapheresis (plasma exchange, PLEX) every other day for 5-10 sessions or intravenous immunoglobulin (IVIG) 0.5 g/kg per day for five days.

Plasma Exchange

PLEX works by removing several antibodies (IgM and IgG), immune complexes, cytokines and other unknown inflammatory mediators [22]. PLEX is widely considered the ultimate treatment choice for autoimmune encephalitis due to its proven success in both steroid and IVIG resistant cases. Plasma exchanges are usually performed every second or the third day and in most cases, it will result in a dramatic improvement after two sessions. Therapy discontinuation can be considered after five to 10 sessions in patients with sustained clinical resolution. Plasma IgG rapidly returns to the pre-apheresis level within 48 hours after PLEX discontinuation due to the production increase from a rebound phenomenon and cellular shifts. Concurrent immunosuppressive therapy (administered after the PLEX session) is important to significantly reduce IgG levels. Angiotensin-converting enzyme (ACE) inhibitor therapy should be withheld 24 hours prior to PLEX to prevent symptoms resembling anaphylaxis (flushing, hypotension, abdominal cramping). Patients should also be monitored closely for the development of bloodstream infections, fluid overload and citrate-induced hypocalcemia. Since PLEX removes any infused IVIG, it should be performed either before or one month after the last dose of IVIG (due to the prolonged effects of the drug).

Intravenous Immunoglobulin

IVIG is produced from pooled plasma donated by several thousand screened donors and has anti-inflammatory and immunomodulating properties that include enhanced clearance of pathogenic IgG [23]. Rapid IVIG infusions should be avoided which can present with anaphylaxis like reactions (chest tightness, wheezing, dyspnea) and volume overload in susceptible patients. True anaphylaxis (hives, angioedema, hypotension and/or respiratory compromise) to IVIG is extremely rare and requires immediate discontinuation of the infusion and administration of intramuscular epinephrine. Delayed (hours to days) reactions that may occur after IVIG infusions include thrombosis (arterial and venous), acute coronary syndrome, transient cerebral ischemia attack, stroke, acute kidney injury, hyponatremia, hemolysis, neutropenia and eczematous dermatitis.

Typical cost of IVIG and PLEX

The typical five-session hospital costs (in US dollars) for both methods of immunomodulation in an 80kg patient will be discussed below (the mentioned prices represent the average hospital drug acquisition costs and patients are typically billed much higher to cover various other expenses). The typical hospital cost of 40 grams IVIG is ~$7,000 and the total five-day cost in an 80kg patient is $35,000. Technical and equipment charges for PLEX cost around $2,000 per session ($10,000 for five). PLEX also requires 5% albumin (each 500mL costs ~$200) and around 4,000mL is required per session in an 80kg patient ($1,600 per session and $8,000 for five). The total PLEX cost in this example is ~$19,000 including the charges for the insertion of a central venous catheter.

Maintenance therapy

The risk of relapse is high in patients with autoimmune encephalopathy and they should be closely monitored while gradually tapering the prednisone dose. Other immunosuppressants (mycophenolate, methotrexate, azathioprine and cyclophosphamide) may be considered if patients develop recurrences while on steroid therapy. Patients may require immunosuppressive therapy between six months to two years based on the clinical response (13). Patients taking chronic steroid therapy should be supplemented with calcium and vitamin D and proton pump inhibitor therapy may be required in patients with a history of gastritis or peptic ulcers. Patients on dual immunosuppression (e.g., steroids plus another agent) also benefit from pneumocystis jiroveci pneumonia prophylaxis with either trimethoprim/sulfamethoxazole or atovaquone.

## Conclusions

Several patients with autoimmune encephalopathy are misdiagnosed with anoxic brain injury and these patients may remain in a stuporous state for several months. While spontaneous recovery can occur, untreated patients with the long-standing diseases often develop cognitive impairment. Our knowledge about autoimmune encephalopathies is rapidly evolving and there are still several unidentified forms, which may still respond to immunomodulating therapy. Normal results of inflammatory markers, MRI, CSF analysis and antibody testing do not always exclude an autoimmune encephalopathy. Since autoimmune and paraneoplastic antibody assays take several weeks to result, clinical response to immunomodulating therapy remains the best way to confirm autoimmune encephalopathy. A poor response to corticosteroid challenge should be followed up with PLEX and IVIG, which are crucial in the successful management of patients with severe autoimmune encephalopathies.
